# Development of third generation anti-EGFRvIII chimeric T cells and EGFRvIII-expressing artificial antigen presenting cells for adoptive cell therapy for glioma

**DOI:** 10.1371/journal.pone.0199414

**Published:** 2018-07-05

**Authors:** Ayguen Sahin, Carlos Sanchez, Szofia Bullain, Peter Waterman, Ralph Weissleder, Bob S. Carter

**Affiliations:** 1 HMS-MGH Center for Nervous System Repair, Department of Neurosurgery, Massachusetts General Hospital, Boston, MA, United States of America; 2 Center for Systems Biology, Massachusetts General Hospital, Boston, MA, United States of America; Mie University Graduate School of Medicine, JAPAN

## Abstract

Glioblastoma multiforme (GBM) is the most aggressive and deadly form of adult brain cancer. Despite of many attempts to identify potential therapies for this disease, including promising cancer immunotherapy approaches, it remains incurable. To address the need of improved persistence, expansion, and optimal antitumor activity of T-cells in the glioma milieu, we have developed an EGFRvIII-specific third generation (G3-EGFRvIII) chimeric antigen receptor (CAR) that expresses both co-stimulatory factors CD28 and OX40 (MR1-CD8TM-CD28-OX40-CD3*ζ*). To enhance *ex vivo* target specific activation and optimize T-cell culturing conditions, we generated artificial antigen presenting cell lines (aAPC) expressing the extracellular and transmembrane domain of EGFRvIII (EGFRVIIIΔ654) with costimulatory molecules including CD32, CD80 and 4-1BBL (EGFRVIIIΔ654 aAPC and CD32-80-137L-EGFRVIIIΔ654 aAPC). We demonstrate that the highest cell growth was achieved when G3-EGFRvIII CAR T-cells were cocultured with both co-stimulatory aAPCs and with exposure to EGFRvIII (CD32-80-137L-EGFRVIIIΔ654 aAPCs) in culturing periods of three to six weeks. G3-EGFRvIII CAR T-cells showed an increased level of IFN-*γ* when cocultured with CD32-80-137L-EGFRVIIIΔ654 aAPCs. Evaluation of G3-EGFRvIII CAR T-cells in an orthotropic human glioma xenograft model demonstrated a prolonged survival of G3-EGFRvIII CAR treated mice compared to control mice. Importantly, we observed survival of G3-EGFRvIII CAR T-cells within the tumor as long as 90 days after implantation in low-dose and single administration, accompanied by a marked tumor stroma demolition. These findings suggest that G3-EGFRvIII CAR cocultured with CD32-80-137L-EGFRVIIIΔ654 aAPCs warrants itself as a potential anti-tumor therapy strategy for glioblastoma.

## Introduction

Glioblastoma multiforme (GBM) or grade IV astrocytoma is the most common and aggressive malignant primary brain tumor in adults. Even after conventional strategies such as surgery and/or chemotherapy the average survival time of a GBM patient is just over 15 months. Its inevitable treatment failure is mainly caused due to its highly invasive and therapy resistant attributes. We and others have previously shown the efficacy of T-cell adoptive immunotherapy for glioblastoma using the CAR (chimeric antigen receptor) technology in preclinical models [1–5], and its safe application is currently being tested clinical studies [6]. Although recent clinical successes with CAR T-cells for CD19+ hematological malignancies have been demonstrated [7], effective clinical applications for solid tumors, including brain tumors, remain challenging and are currently under extensive investigation. CARs directly recognize cell surface antigen in an MHC-independent manner, making them universal for all patients and resistant to tumor escape by MHC downregulation. Careful selection of the target antigen is one of the key factors in CAR T-cell-based immunotherapy strategies as targeting molecules on solid tumors that are not strictly tumor specific may retain significant potential for on-target, off-tumor toxicities, such as ERBB2/ HER2 [8]. The majority of GBMs exhibit a frequent genetic alteration, EGFR amplification, and a subset of this alteration contains the mutant EGFR gene, EGFRvIII [9]. Up to 30% of GBM specimens express EGFRvIII [9]. The presence of EGFRvIII mutation increases glioma proliferation, invasion [10, 11], and therapeutic resistance [12]. On the other hand, EGFRvIII represents an ideal therapeutic target as it is not expressed in normal brain tissue [13]. Our group has focused on CAR T-cell immunotherapy for glioblastoma specifically directed to target EGFRvIII. We and others have previously shown EGFRvIII to be a promising target for gene-modified CAR T-cell therapy for gliomas both *in vitro* and *in vivo* models [2, 4, 13–16]. Genetically modified T-cells re-directed to recognize EGFRvIII and other targets such as IL13R2 or HER2 are currently being assessed for safety and efficacy in clinical studies for glioblastoma ([6], Clinicaltrials.gov: NCT01454596, Clinicaltrials.gov: NCT01109095, Clinicaltrials.gov: NCT02208362). In this study we have adapted our previously reported plasmid based transfection of a first generation EGFRvIII-specific CAR and developed a ‘third generation’ EGFRvIII CAR, incorporating the intracellular costimulatory domains of CD28 and OX40 in addition to CD3*ζ* signaling. ‘Third generation’ CARs have shown benefits in preclinical settings over ‘second generation’ CARs, which typically incorporate CD28 or 4-1BB (CD137) to enhance CAR T-cell function via increased cytokine production, T-cell proliferation, and killing in the setting of prior exposure to antigen [17]. For example, in third generation CARs, costimulatory molecules such as OX40 provide benefits with respect to activation and persistence of both CD4 and CD8 T-cells [18–21]. To assess the best culture conditions for short-term and long-term propagation of this third generation EGFRvIII CAR approach and to test whether its antigen-specific activity can be enhanced, we also developed artificial antigen presenting cell lines (EGFRVIIIΔ654 aAPC and CD32-80-137L-EGFRVIIIΔ654 aAPC), that express EGFRvIII on its cell surface (lacking its intracellular domain). Here, we report here that assessments of both *in vitro* cytolysis of EGFRvIII target tumor cells as well as improved survival in an EGFRvIII positive intracranial human glioblastoma xenograft mouse model provide encouraging data that shows ‘third generation’ EGFRvIII-specific CAR T-cells, cocultured with EGFRvIII-specific aAPCs that additionally express CD32, CD80, and CD137L (4-1BBL) co-stimulatory molecules presents itself to be an effective strategy for preparation of EGFRvIII directed CAR therapy for human glioma.

## Materials and methods

### Creation of third-generation anti-EGFRvIII CAR and EGFRvIII expressing artificial antigen presenting cell lines (aAPCs)

**Fig 1:**
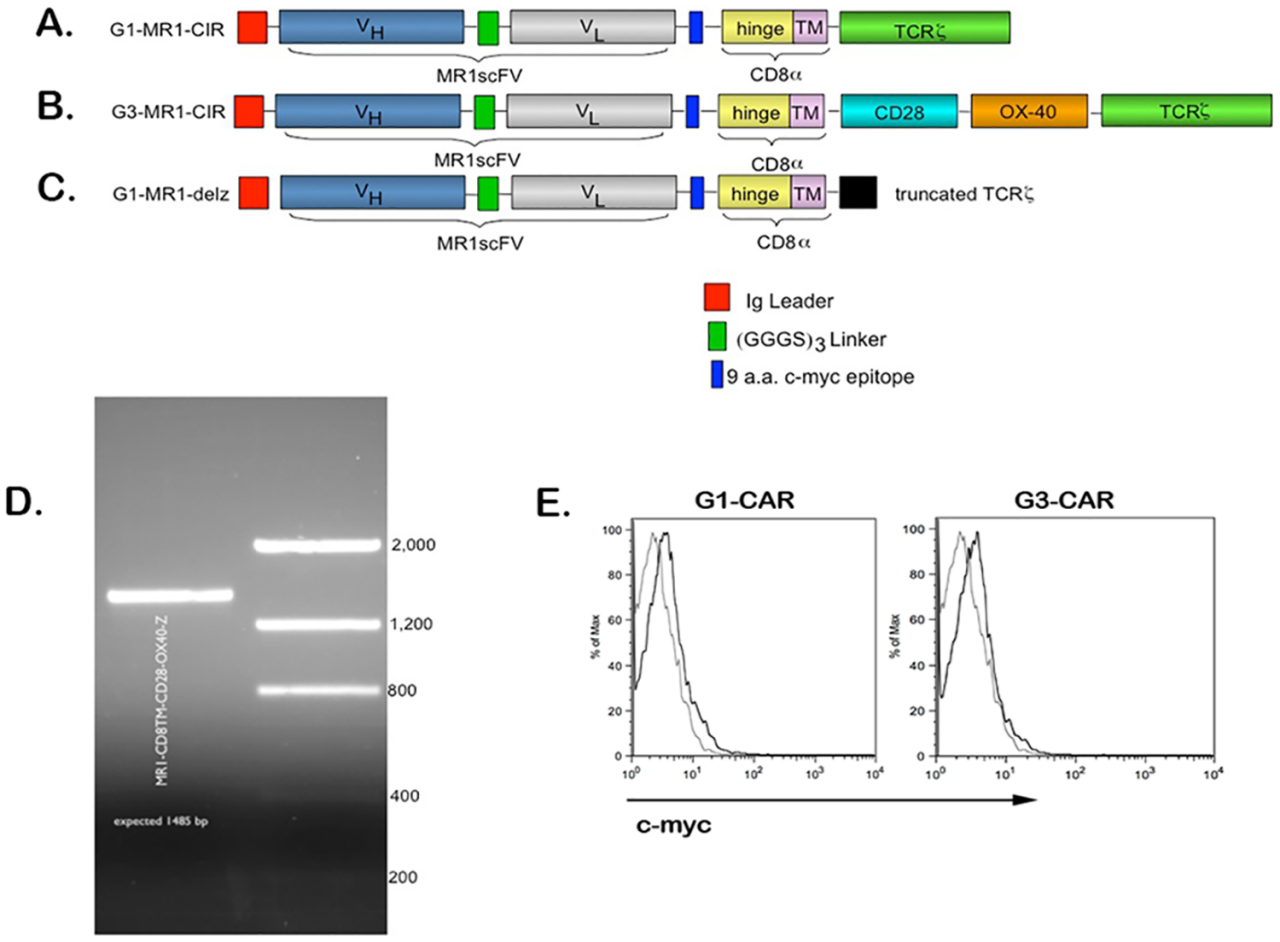
G3-EGFRvIII CAR expression, cell surface trafficking, and artificial antigen presenting cells. (A)Schematic representation of G3-EGFRvIII CAR along with G1-EGFRvIII CAR, and del*ζ*-EGFRvIII CAR (partially deleted TCR). The MR1-scFv was coupled with a CD8 hinge and the CD8 transmembrane TM domain. (B)CD28 and OX40 signaling domains were incorporated between CD8 TM domain and TCR signaling domains. A 9 a.a. c-myc epitope was incorporated between scFv and CD8 hinge and CD8 TM regions. (C)A deletion mutant confers lower signaling capacity in the del*ζ* mutant. (D)Verification of G3-EGFRvIII CAR (MR1-CD8TM-CD20-OX40-CD3) by RT-PCR amplification. (E)Flow cytometric detection of cell surface G1-EGFRvIII CAR (G1) or G3-EGFRvIII CAR (G3) on PBMC transfectants 9 days post nucleofection.

We have previously described [2] the construction of a first generation (G1) anti-EGFRvIII CAR (MR1-CD3*ζ*; Fig 1A). A third generation (G3) anti-EGFRvIII CAR (MR1-CD8TM-CD28-OX40-CD3*ζ*; Fig 1B) was created by incorporating the CD28 intracellular domain and the OX40 signaling domain between the CD8 alpha transmembrane region and the CD3-*ζ* molecule of the original pMG-MR1-*ζ* backbone. A 9 amino acid c-myc epitope was incorporated in this backbone between scFv, and a CD8 hinge and CD8 transmembrane region. The original pMG-MR1-*ζ* backbone was designed to co-express hygromycin phosphotransferase-HSV thymidine kinase (HyTK) selection/suicide fusion gene as described previously [2]. CD28 and OX40 fragments were amplified by PCR using suitably designed primers assembled in between the construct by restriction digestion. The final 1485 base pairs (bp) DNA fragment of G3-MR1 CAR encoding product was amplified with specific primers (forward, 5’-GCCAGACTAGTGACAAGAGGCTGG-3’, and reverse, 5’-GGGCCGCTCAGCGAGGGGGCAGGG-3’) at a concentration of 10 *μ*M, and was verified by sequencing. We have also previously described the production of a signaling defective version of MR1-CD3*ζ* (MR1-CD3del*ζ*; Fig 1C) [2].

### Cell lines

The U87vIIIffluc target cell line was generated in our laboratory as previously described [2, 22]. The cell line were maintained in Minimal Essential Medium Eagle’s (ATCC), supplemented with 10% fetal bovine serum and 1% of 10,000 IU/ml penicillin/10,000*μ*g/ml streptomycin. To determine the specificity and activity of G3-EGFRvIII CAR T-cells we used the human glioblastoma U87vIIIffluc line [2] as target cells throughout this study. To confirm firefly luciferase expression, U87vIIIffluc target cells were incubated overnight on microscopic cover slips. Cells were then fixed with 4% PFA in PBS. Unspecific binding was eliminated using blocking solution prior to staining with rabbit anti-firefly luciferase [L0159] polyclonal antibody (1:200, Sigma-Aldrich, St. Louis, MO). Subsequently, cells were stained with Alexa Fluor488 goat anti-rabbit IgG (H+L), highly cross-absorbed, 2mg/ml (1:1500, Invitrogen). Finally, cells were counter stained with 4,6-diamidino-2-phenylindole (DAPI), and examined under immunofluorscence microscope (Leica Microsystems). EGFRvIII expression in the U87vIIIffluc line was detected by staining with L8A4 mouse monoclonal antibody (generous gift of Dr. Darell Bigner, Duke University), and then followed by R-phycoerythrin (R-PE) conjugated rat anti-mouse monoclonal antibodies (BD Pharmingen). EGFRvIII and firefly luciferase expressions of U87vIIIffluc cells were determined as 97.5% and 100%, respectively prior to initiating experiments. (Data not shown).

**Fig 2:**
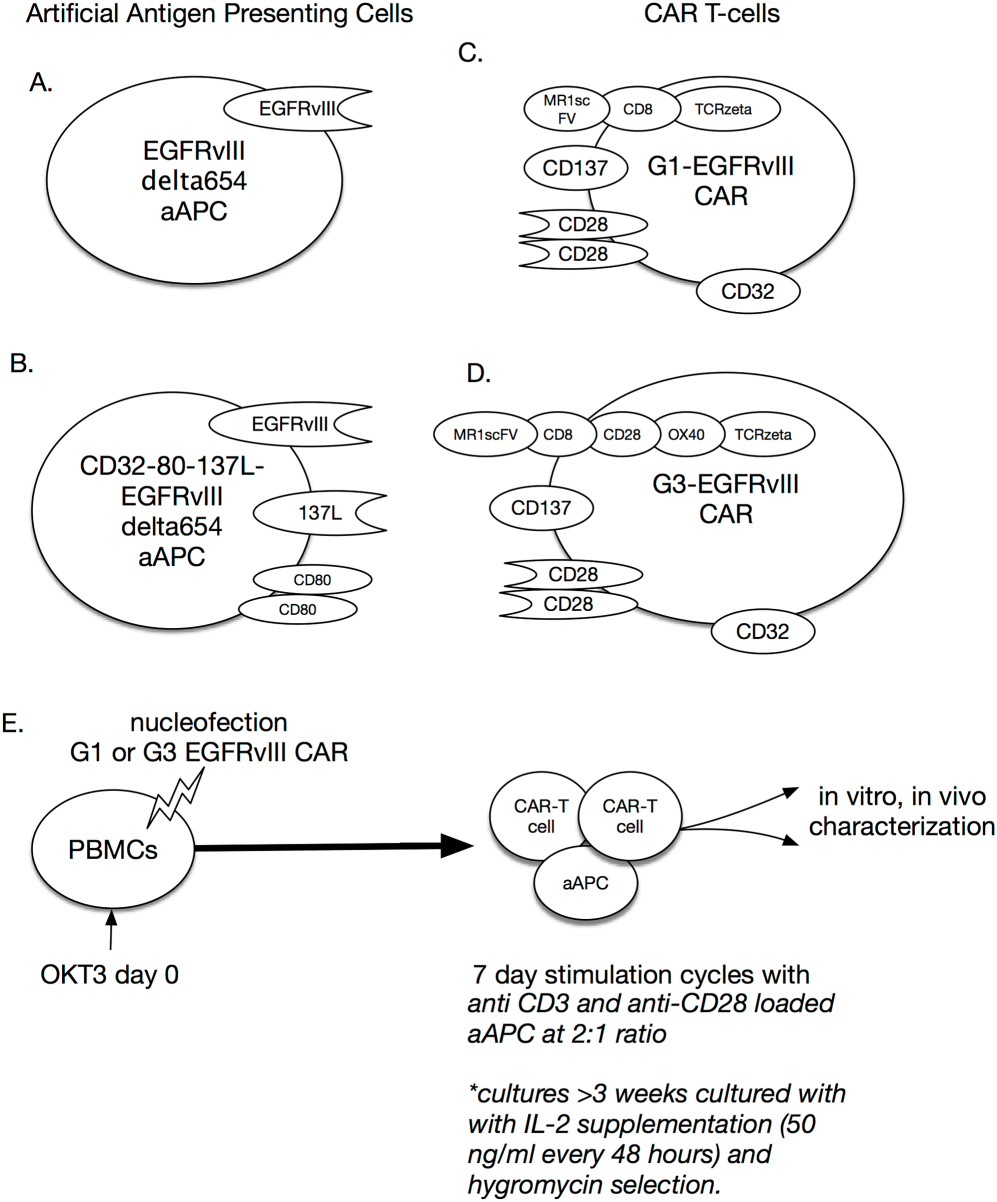
Artificial antigen presenting cells (aAPCs). (A)aAPC based on the K562 cell line encoding a non-signaling EGFRvIII mutant. (B)aAPC based on the K562 cell line encoding a non-signaling EGFRvIII mutant in addition to CD32, CD80, and CD137L. (C)G1-EGFRvIII CAR (D)G3-EGFRvIII CAR (E)Overall transduction and stimulation protocol for (F)three week and (G)six week (under selection) of CAR T-cells.

Two new artifical antigen presenting cell lines (aAPCs) were created for this study: K562 cells expressing the extracellular domain of EGFRvIII (EGFRVIIIΔ654 aAPC; Fig 2A.), and a hybrid K562 cell line expressing EGFRvIII and the costimulatory antigens CD32-80-137L (CD32-80-137L-EGFRVIIIΔ654 aAPC; Fig 2B) based on K562 cells expressing CD32-80-137L aAPCs, which were a generous gift of Carl June, University of Pennsylvania.

For the construction of EGFRvIII extracellular domain expressing aAPCs, the human EGFRvIII fragment was amplified from the pBABEpuro-EGFRvIII construct (a gift of Dr. Miguel Esteves, Massachusetts General Hospital). The EGFRvIIIΔ654 construct was generated by replacing the first intracellular codon at 654 with a TGA stop codon. The EGFRvIIIΔ654 molecule was assembled in a pHAGE-CMV lentiviral vector. The construct was verified by sequencing. Lentivirus was generated through cotransfection of lentiviral construct with gag/pol, rev, and vsv.g plasmids into packaging cell line HEK 293T-cells. Resultant lentivirus was used to infect K562 or K562-CD32-CD80-137L cell lines with 8*μ*g/ml polybrene. EGFRVIIIΔ654 and CD32-80-137L-EGFRVIIIΔ654 aAPCs aAPC cells were created by transduction of K562 cells with the lentiviral vector EGFRvIIIΔ654, described above. The genetically engineered CD32-80-137L-EGFRVIIIΔ654 aAPC or EGFRVIIIΔ654 aAPC cells were either FACS sorted for high EGFRvIII expression, or high EGFRvIII expressing cells were clonally expanded using limiting dilution method in 96 well plates. CD32-80-137L aAPC cells were cultured in AIMV media (Gibco BRL/Life Technologies), supplemented with 3% human AB serum (Valley Biomedical), as described before [23]. CD32-80-137L-EGFRVIIIΔ654 aAPC or EGFRVIIIΔ654 aAPC cells were cultured in Iscove’s Modified Dulbecco’s Medium (ATCC), supplemented with 10% fetal bovine serum. CD32-80-137L-EGFRVIIIΔ654 and EGFRVIIIΔ654 aAPCs were stained for EGFRvIII expression as described above, and sorted on FACSAria (BD Biosciences). Single EGFRVIIIΔ654 aAPC cells were expanded using the ‘cloning by limiting dilution’ method. Cell suspension was serial diluted with media, to the extent that each 96-well plate contained one cell. Each single cell clone was expanded, stained for EGFRvIII expression, and analyzed using flow cytometry. To detect the expression of costimulatory molecules on CD32-80-137L aAPCs and CD32-80-137L-EGFRVIIIΔ654 aAPCs, cells were triple-stained with Phycoerythrin-conjugated mouse monoclonal [AT10] anti-human CD32 (Abcam, Cambridge, MA), Phycoerythrin-Cy5 (PE-Cy5)-conjugated mouse monoclonal [2D10.4] anti-human CD80 (eBioscience) and CD137L (41BBL, Medical&Biological Laboratories, Woburn, MA). T-cells were stained with PE-conjugated c-myc [9E10] monoclonal antibody (Santa Cruz Biotechnology) to detect the CAR expression. In all cases, cells were stained with antibodies at 4°C and analyzed on GuavaEasyCyte^™^ (Guava Technologies, Inc. Hayward, CA) or LSR II (BD Biosciences). All flow cytometry data were analyzed with FlowJo software (Tree Star, Ashland, OR).

### Nucleofection, polyclonal stimulation and long-term culture of CAR T-cells

Human peripheral blood mononuclear cells (PBMCs) were obtained from healthy donors via Ficoll gradient separation. PBMCs were nucleofected with MR1-CD3*ζ*, MR1-CD28-OX40-CD3*ζ*, MR1-del*ζ* CAR, or PMG-GFP (GFP) plasmid DNA to create a population of G1-EGFRvIII CARs (MR1-CD3*ζ*; Fig 2C), G3-EGFRvIII CARs (MR1-CD28-OX40-CD3*ζ*; Fig 2D) or controls. Nucleofection was performed according to the manufacturer’s protocol with slight modifications (Nucleofector I., Amaxa Biosystems). Briefly, 60-80 million freshly isolated PBMCs per group were divided into 20 million cells and nucleofected with 4-8 *μ*g plasmid DNA. Nucleofected cells were then cultured in Roswell Park Memorial Institute-1640 (Gibco/Invitrogen) supplemented with 10% heat inactivated fetal bovine serum (HyClone, Logan, UT) and 1% 10,0000 U/ml penicillin and 10,000 *μ*g/ml streptomycin (HyClone), and incubated at 37°C in 5%CO2. Six hours after nucleofection, T-cells were activated by 30ng/ml OKT-3 (a monoclonal antibody against human CD3, Ortho Biotech) on day 0. Fifty IU/ml recombinant human IL-2 (Chiron) was added to the cultures every other day starting on day 1. Starting on day 3, every other day, cells were selected on hygromycin B (Invivogen) to maintain the gene expression as described previously [2]. The first stimulation cycle began on day 7 and repeated in 7-day cycles. Before stimulation at a 2:1 (CAR T-cell:aAPC) ratio, CD32-80-137L aAPC, CD32-80-137L-EGFRVIIIΔ654 aAPC or EGFRVIIIΔ654 aAPC cells were lethally irradiated with 100 Gy, washed, and resuspended at 1x10^6^ cells/ml in T-cell media. CD32-80-137L aAPC and CD32-80-137L-EGFRVIIIΔ654 aAPCs were added to a 12 or 24 well plate and were loaded with 0.5ug/ml mouse monoclonal anti-human CD3 (OKT3, Ortho Clone) and mouse monoclonal anti-human anti-CD28 [CD28.2] (BD Pharmingen) for 10 min at room temperature. T-cells were resuspended at 2x10^6^ cells/ml and added in a 0.5 ml volume drop-wise to the antibody-loaded aAPCs. T-cell cultures were monitored and counted every 2-3 days, and were restimulated when cell growth leveled.

### Biophotonic cytolytic assay

Luciferase based biophotonic cytolytic assay was performed as described previously [24]. Briefly, 7 days after nucleofection of PBMCs with MR1-CD3*ζ*, MR1-CD28-OX40-CD3*ζ*, MR1-del*ζ* CAR plamids, cells were added to U87vIIIffluc target cells in a V-bottom microplate at a 25:1 (effector:target) ratio. Long-term cultured (six restimulation cycles) cell groups were added to 50,000 U87vIIIffluc cells at 5:1, or 10:1 ratios. Luminescence signal was read in counts per second (CPS) after incubation time for 5 hrs using a VICTOR3^™^ Multilabel Plate Reader (PerkinElmer Inc.). Percentage of specific lysis was calculated by a mathematical formula as described previously [24].

### Cytokine production

Freshly harvested PBMCs were nucleofected with MR1-CD3*ζ*, MR1-CD28-OX40-CD3*ζ*, MR1-del*ζ* CAR plamids and cocultured with CD32-80-137L aAPC, CD32-80-137L-EGFRVIIIΔ654 aAPC, or EGFRVIIIΔ654 aAPC at 7-day cycles for 3 restimulation cycles. Cells were then added to 50,000 U87vIIIffluc cells at a 25:1 ratio in a V-bottom 96 well microplate. Supernatants were collected 24 hours thereafter and were snap frozen in liquid nitrogen and then stored in -80°C until the time of experimental use. IL-2 and IFN-*γ* production was measured by enzyme-linked immunosorbent assay (ELISA) using the Human Th1/Th2 ELISA Ready-SET-Go! kit (eBioscence), according to the manufacturer’s protocol.

### Treatment of glioblastoma in NOD/SCID mice and imaging

Six to eight week-old, female, NOD/NCrCrl-PrkdcSCID mice were obtained from Charles River Laboratories. All animals were housed in specific pathogen free environment and were strictly kept under the accordance of protocols approved by the Institutional Animal Care and Use Committee (IACUC) of Massachusetts General Hospital (Permit Number: 2005N000250), with standard animal care requirements. All efforts were made to minimize suffering. On experimental day 0, mice underwent intracranial (i.c.) implantation of 25,000 U87vIIIffluc target and 25,000 effector cells (G1-EGFRvIII CAR, G3-EGFRvIII CAR, or GFP-T cells) in 2 *μ*l T-cell media using stereotactic coordinates (0.5 mm posterior, 2.5 mm lateral, and 3.5 mm intraparenchymal from the bregma). Target and effector cells were mixed in a separate microcentrifuge tube immediately before each implantation. Tumor progression was followed by bioluminescence imaging (BLI) as previously described [25] every 3–5 days. The weight of the mice was monitored at daily and with each imaging session to assure animal health and lack of neurologic toxicity and death was recorded. Mice in remission were followed for recurrence of disease for 121 days were then euthanized for further histological analysis. No IL-2 was administered during the course of the experiment. Briefly, the mice were anesthetized using 1-2% isoflurane during intraperitoneal injection (i.p.) of D-luciferin (150mg/kg in 200*μ*l PBS; Caliper Life Sciences) 10 min prior to imaging. Bioluminescence imaging was performed in a high sensitivity, cooled CCD camera (IVIS^®^, Xenogen) at five-minute intervals until peak emission value was obtained and a decrease in emission was observed for all mice. Living image^®^software (Xenogen) was used for image capture and signal quantification. Kaplan-Meier survival curve was generated using Prism software (Prism 5.0a, Graphpad Software).

### Immunohistological staining

Animals were deeply anesthetized and perfused in the left ventricle with chilled phosphate buffered saline (PBS) followed by chilled 4% paraformaldehyde (PFA) in 1xPBS. The brain was extracted and post-fixed for 12 hours in chilled 4% PFA at 4°C before being paraffin embedded. 5 micrometer coronal sections were incubated in blocking solution (10% normal goat serum and 0.3% TritonX-100 in PBS). Following protein block, sections were incubated with rabbit monoclonal anti-human CD3 antibody [CME324A] (1:400, Biocare Medical) at room temperature for 2 hrs. Subsequently, sections were incubated with Alexa Fluor488 goat anti-rabbit IgG (H+L), highly cross-absorbed, 2mg/ml (1:1500, Invitrogen) for 45 min. Finally, sections were lightly counter-stained with DAPI for 5 minutes. For negative control, incubation step with primary antibody was omitted. Immunofluorescent microscopy was carried out using a Leica confocal microscope (Leica Microsystems). For anatomical evaluation, every tenth of serial sectioned brains were stained with hematoxylin and eosin (H&E). Images were visualized under an optical microscope at 20x, 100x and 200x magnification.

### Statistical analysis

Group comparison of mean expression levels were compared by F test on untransformed raw data. Significance was at the .05 level.

## Results

### CAR construction and expression

To construct a third generation anti-EGFRvIII CAR, we linked the anti-EGFRvIII scFv with a partial extracellular domain (hinge) and transmembrane domain (TM) of CD8*α*, and coupled the CD28 and OX40 intracellular domains with the cytoplasmic domain of human CD3*ζ*. Fig 1A–1C demonstrate a schematic description of the G3 versus the G1 and del*ζ*-EGFRvIII CARs and the confirmed RNA expression of the G3 construct (Fig 1D) in transduced human T-cells. By cell surface protein staining with an anti-myc antibody, we found that the myc tag just proximal to the hinge region in the CAR was typically detected as low magnitude population shift of mean fluorescence of 0.5 log units (two different donors, Fig 1C).

### Creation of EGFRvIII specific aAPCs

To test the target-specific activation of G3-EGFRvIII CAR T-cells, and optimum CAR T-cell expansion conditions *in vitro*, we generated two artificial APC lines: EGFRVIIIΔ654, and CD32-80-137L-EGFRVIIIΔ654 aAPC (Fig 2A and 2B). The expression of costimulatory molecules were determined by triple antibody staining and flow cytometric analysis, resulted in 99.8% for CD80, and 93.5% for CD137L in CD32-80-137L aAPC cells, and 99.6% for CD80, and 97.3% for CD137L in CD32-80-137L-EGFRVIIIΔ654 aAPC cells (data not shown). We selected EGFRvIII expressing CD32-80-137L-EGFRVIIIΔ654 aAPC or EGFRVIIIΔ654 aAPC cells using FACS sorting or cell cloning through serial dilution method, respectively. After cell expansion, expression of EGFRvIII in CD32-80-137L-EGFRVIIIΔ654 aAPCs and EGFRVIIIΔ654 aAPCs was 53.2% and 69.7%, respectively, as determined by flow cytometric analysis (data not shown).

### Transduced G3-EGFRvIII CAR T-cells demonstrate rapid short-term cell expansion when exposed to EGFRvIII positive aAPCs

**Fig 3:**
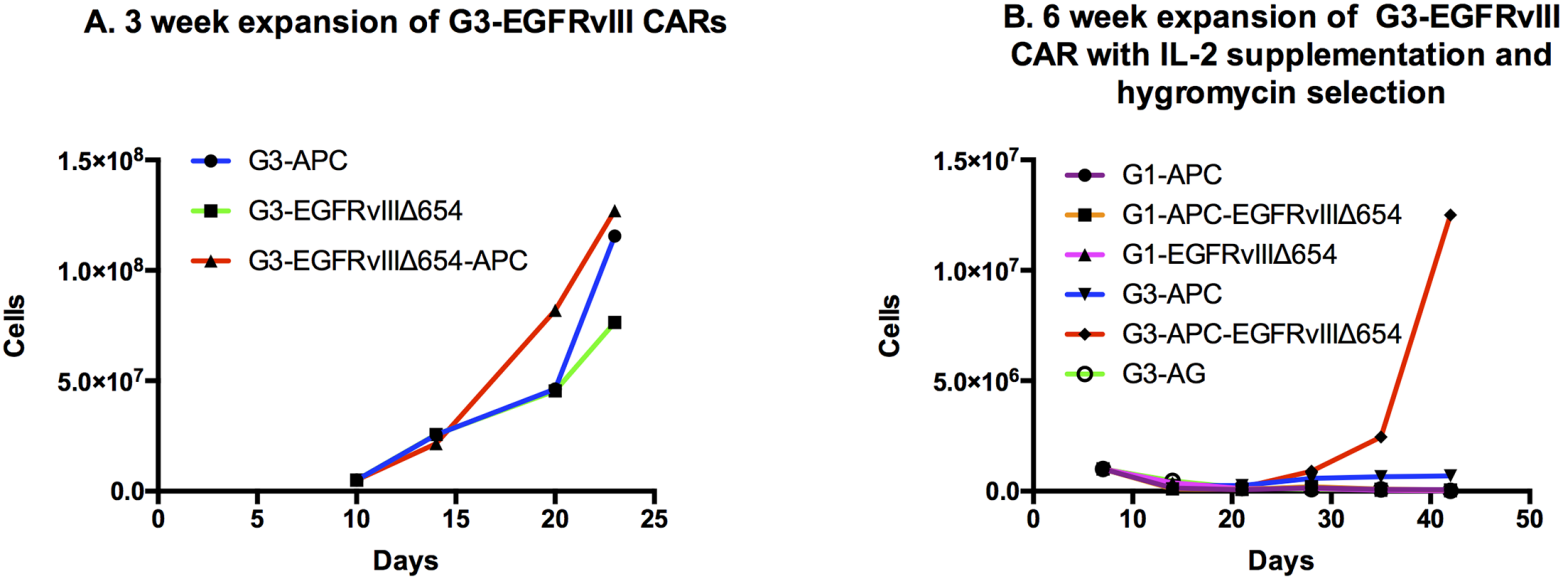
CAR T-cell expansion with and without selective conditions. (A)Short-term culture. G3 CAR T-cells were co-cultured with three different aAPCs as shown. (B)G1 vs G3 CAR T-cells grown for 6 restimulation cycles under hygromycin selection.

We next carried out experiments to determine efficiencies of feeder cell lines with respect to G3-EGFRvIII CAR T-cell expansion in short-term cultures (<21 days) and in medium to long-term cultures (>21 days) with aAPC restimulation and hygromycin selection. In short-term culture conditions of three weeks, G3-EGFRvIII CAR T-cells were cocultured with irradiated CD32-80-137L aAPCs, CD32-80-137L-EGFRVIIIΔ654 aAPCs (100 G irradiation), EGFRVIIIΔ654 aAPCs, at a 2:1 (CAR T-cell: aAPC) ratio. In short-term culture, G3-CAR T-cells underwent a robust 15-25 fold expansion of cells in a three week culturing period (Fig 3). G3-EGFRvIII CAR T-cell expansion was highest (from 5E6→1.27E8) when cocultured with CD32-80-137L-EGFRVIIIΔ654 aAPC, followed by CD32-80-137L aAPC (5E6→1.16E8) compared to EGFRVIIIΔ654 aAPC (7.64x10^7^). This result suggests that for short-term expansion, G3-EGFRvIII CARs are suitably expanded by exposure to aAPCs that express EGFRvIII antigen, however, that the addition of an aAPC phenotype that includes cells surface expression of CD32, CD80, and CD137L enhances this expansion several fold.

### G3-EGFRvIII CAR T-cells co-cultured with EGFRvIII-positive aAPCs demonstrate improved expansion under long-term selection versus G1-EGFRvIII CARs

To assess G3-EGFRvIII CAR T-cell proliferation under more demanding long-term culture conditions that included both IL-2 supplementation and hygromycin selection (to maximize retention of the EGFRvIII CAR construct within the population), we co-cultured the G3-EGFRvIII CAR, with various types aAPCs using restimulation cycles every 7 days at ratio of 2:1 (EGFRvIII CAR: aAPC) for up to six weeks. We also compare the G3 long-term culture response to our previously described G1 CARs (G1-EGFRvIII and G1-EGFRvIII-del*ζ*) as controls. In the context of hygromycin selection, we found that exponential growth was achieved in the G3-EGFRvIII CAR + CD32-80-137L-EGFRVIIIΔ654 aAPC group (1.25x10^7^, 12 fold expansion, Fig 3B) after six restimulation cycles. In contrast to the prior finding of robust short-term expansion, we did not observe exponential expansion of the G3-EGFRvIII CARs when co-cultured to CD32-80-137L aAPCs or EGFRVIIIΔ654 aAPCs. This suggested that long-term culturing under these conditions required both a third Generation CAR as well as an aAPC engineered to provide both antigen specific stimulation as well as additional cell surface co-stimulator molecules.

### G3-EGFRvIII CAR T-cells maintain comparable cytotoxicity to G1-EGFRvIII CARs and preserve their cytotoxic ability after long term culture

**Fig 4:**
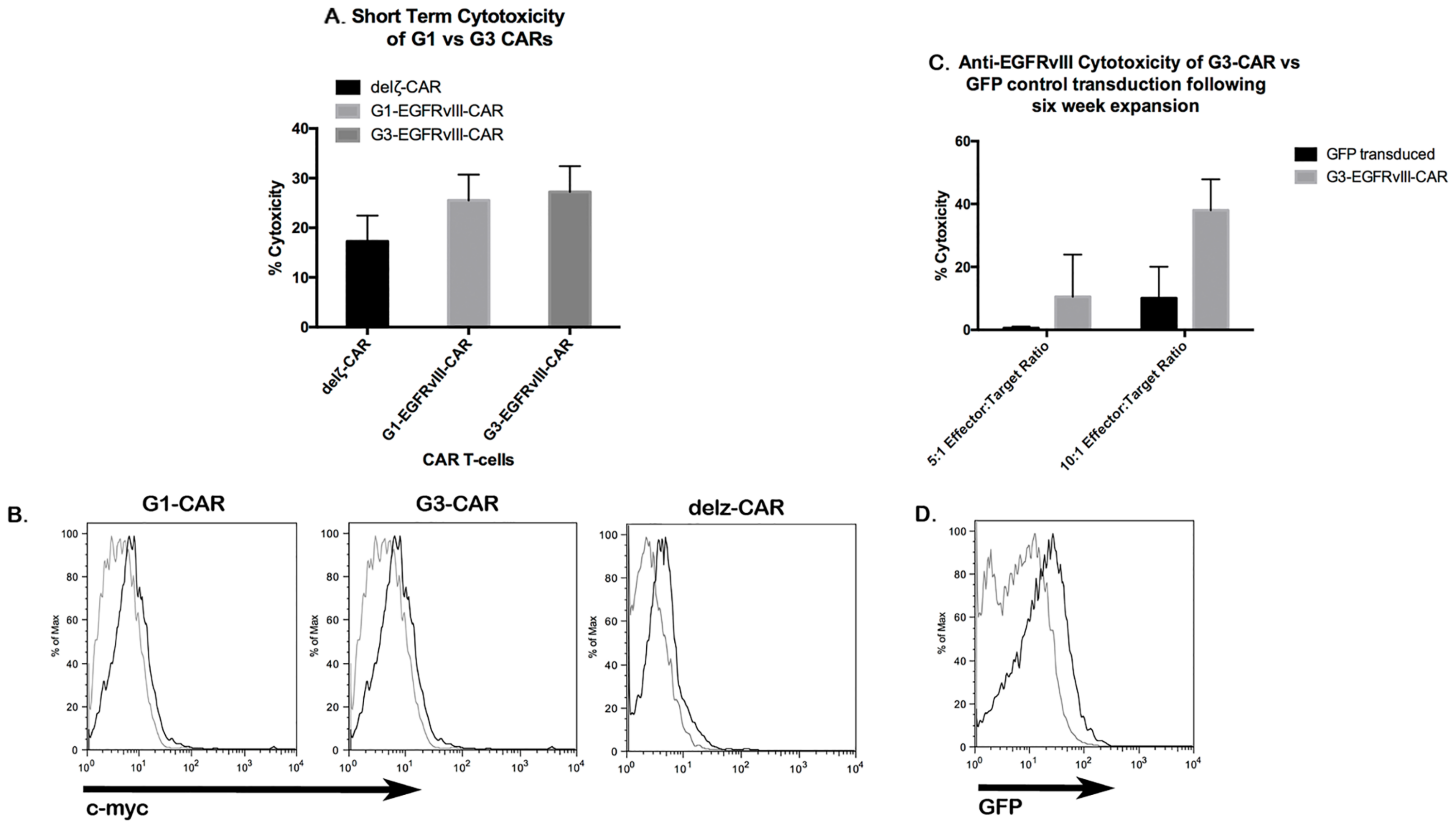
G1 vs G3 CAR and durability of cytoxicity in culture. (A)Short term biophotonic cytolysis assay. Seven days after nucleofection of PBMCs with G1-EGFRvIII CAR (G1), G3-EGFRvIII CAR (G3), or del*ζ*-EGFRvIII CAR [2] plasmids, cells were added to U87vIIIffluc cells at a 25:1 ratio. 5 hours after incubation, T-cell activity was measured by bioluminescence release, and percentage of specific release was calculated for each group. Mean cytolysis values ± SEM is shown for n = 3 for all groups. (B)Flow cytometric detection of G1-EGFRvIII, G3-EGFRvIII, or delζ-EGFRvIII CAR cell surface expression on PBMC transfectants 7 days post nucleofection. Expression of c-myc was typically detected as low magnitude population shift of mean fluorescence of 0.5 log units. (C)Post-culturing cytolysis in PBMCs that were nucleofected with G3-MR1 plasmids and cocultured with irradiated CD32-80-137L-EGFRVIIIΔ654 for 6 restimulation cycles (7-day cycles). Cells were added to U87vIIIfluc target cells at 5:1 or 10:1 ratios. Mean cytolysis values ± SEM is shown for n = 3 for all groups. (D)Flow cytometric detection of GFP in control PMG-GFP-T cells following six-week expansion. GFP expression was observed as a low fluorescence intensity shift of the entire transduced population versus control cells.

A biophotonic bioluminescence assay was performed to determine whether G3-EGFRvIII CAR was effective in recognizing and lysing U87vIIIffluc target cells as compared to the G1-EGFRvIII CAR construct, which has been previously validated [2]. After a 5-hour co-incubation of EGFRvIII CARs with U87-EGFRvIII positive cells (25:1 ratio), a biophotonic assay for cytolysis demonstrated a similar specific cytotoxicity value of G3-EGFRvIII CAR T-cells with G1-EGFRvIII CAR T-cells (25.5% and 27.2%, respectively, Fig 4A), both of which were higher than an attenuated del*ζ*-EGFRvIII CAR (Fig 4A). The del*ζ*-EGFRvIII CAR still permits binding to EGFRvIII but has defective signaling a the level of the CAR. The comparison of CAR constructs here confirmed that the G3-EGFRvIII CAR T maintained a similar cytotoxicity as had been observed with our prior G1-EGFRvIII CAR. The c-myc tag was detected as low magnitude population shift of mean fluorescence of 0.5 log units (two different donors, Fig 4B).

To assess whether long-term culture with several restimulation cycles would impact the cytotoxic activity of the G3-EGFRvIII CAR, we performed bioluminescence cytotoxic assay with CARs stimulated with G3/CD32-80-137L-EGFRVIIIΔ654 aAPC after the 6th restimulation. In a cytoxic assay versus U87vIIIffluc target cells in 5:1 or 10:1 (effector:target) ratios, we observed a somewhat enhanced 38% specific cytotoxicity at the 10:1 ratio, which compared favorably with the results of short-term cultures (Fig 4C). Under constant hygromycin selection pressure in long-term culture for 6 restimulation cycles, GFP expression was observed as a low fluorescence intensity shift of the entire transduced population versus control cells (Fig 4D).

### Improved IFN-*γ* production by G3-EGFRvIII CAR T-cells compared to G1-EGFRvIII and del*ζ* CAR T-cells

To determine antigen-specific cytokine secretion of G3-EGFRvIII CAR T-cells after co-culturing with CD32-80-137L, CD32-80-137L-EGFRVIIIΔ654, or EGFRVIIIΔ654 aAPCs, ELISA assay was performed from supernatants collected 24h post-cytolysis assay to assess IFN-*γ* and IL-2 expression levels.

**Fig 5:**
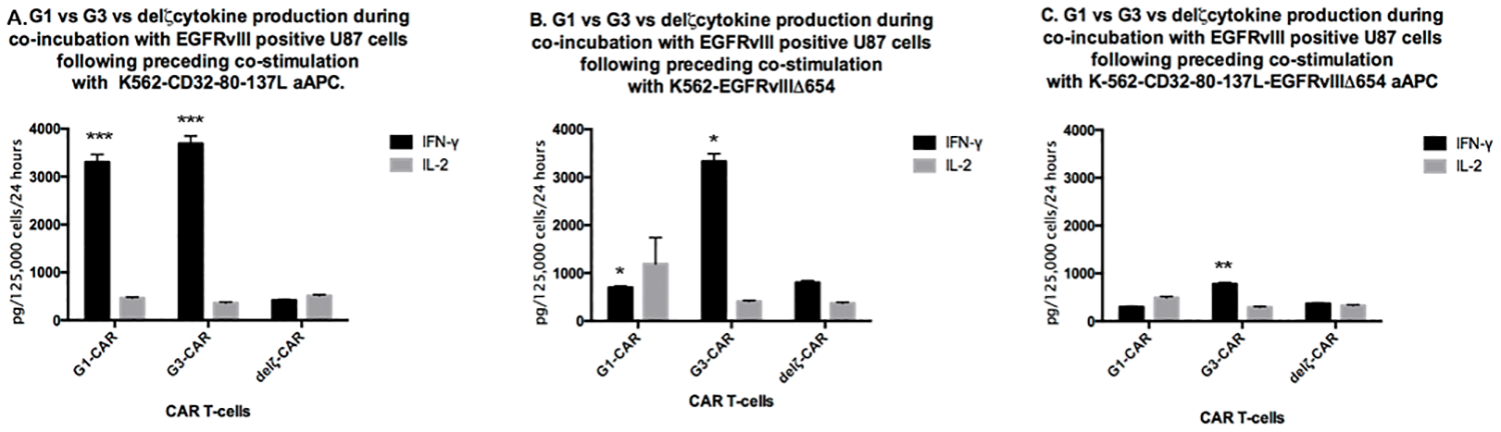
Comparison of CAR T-cell cytokine expression by CAR T cells cocultured with different feeder cell lines. G1 (1x10^6^), G3 (1x10^6^), or del*ζ* (8x10^4^)-T-cells were stimulated in 7-day cycles with irradiated CD32-80-137L (A. left panel), CD32-80-137L-EGFRVIIIΔ654 (B. middle panel) (both preloaded with anti-CD3, and anti-CD28), or EGFRVIIIΔ654aAPCs (C. right panel) at a 2:1 ratio. CAR T-cells were then exposed to U87-EGFRvIII tumor cells and supernatants were collected 24 hours thereafter. IFN-*γ* (solid black bar), and IL-2 (grey bar) expression was measured by ELISA. Significant difference in IFN-*γ* expression was observed in G3 CAR T-cells when cocultured with CD32-80-137L aAPC compared to delζ CAR T-cells (F_2,2_ = 8717, *p* = .0002), as well as in G1 CAR-T cells compared to del*ζ* CAR T-cells [(*F*_2,2_ = 8157, *p* = .0002), A]. When cocultured with EGFRVIII654 aAPC, G3 CAR T-cells demonstrated significantly robust IFN-*γ* expression compared to G1 (*F*_2,2_ = 91.47, *p* = .0216) and del*ζ* CAR T-cells [(*F*_2,2_ = 98.59, *p* = .0201), B]. Significant IFN-*γ* production was observed when G3 CAR-T cells were cocultured with highly modified CD32-80-137L-EGFRVIII654 aAPC compared to G1 CAR-T cells [(*F*_2,2_ = 1194, *p* = .0017), **C**]. No exogenous IL-2 was utilized during the preceding restimulation cycles or during the co-incubation with tumor cells. Mean cytokine expression values ±SEM is shown for n = 3 for all groups. Asterisks indicate significant differences (***, *P* ≤ 0.0001; **, *P* ≤ 0.001, *, *P* ≤ 0.01, see Results).

When pre-cultured on CD32-80-137L aAPC (Fig 5A), both G1 (*F*_2,2_ = 8157, *p* = .0002) and G3 CAR T-cells (*F*_2,2_ = 8717, *p* = .0002)showed robust IFN-*γ* expression (>3000 pg/125,000 cells/24 hours) compared to del*ζ* CAR T-cells upon co-incubation with EGFRvIII positive tumor cells in a cytolysis assay. In contrast, when only antigen was present on the APCs without other costimulatory molecules (EGFRVIIIΔ654 aAPC; Fig 5B), only the G3 CAR T-cells demonstrated significantly robust IFN-*γ* expression of >3000 pg/125,000 cells/24 hours compared to G1 (*F*_2,2_ = 91.47, *p* = .0216) and *del* *ζ*ζ CAR T-cells (*F*_2,2_ = 98.59, *p* = .0201). When the highly modified CD32-80-137L-EGFRVIIIΔ654 aAPC (Fig 5C) served as the pre-cytolysis co-cultured aAPCs, the overall levels of IFN-*γ* production were less than <1000 pg/125,000 cells/24 hours for all CARs, though the G3 CAR-T cells demonstrated the highest level of IFN-*γ* production by two-fold over the G1 (*F*_2,2_ = 1194, *p* = .0017) or del*ζ* CAR T-cells.

When we assessed IL-2 expression levels (Fig 5), we found the expression levels not at substantial levels, and no differences were observed within G1-EGFRvIII CAR, G3-EGFRvIII CAR, del*ζ*-EGFRvIII CAR T-cells aAPCs groups.

### G3-EGFRvIII CAR T-cells extend survival of tumor bearing mice and destroy tumor tissue

**Fig 6:**
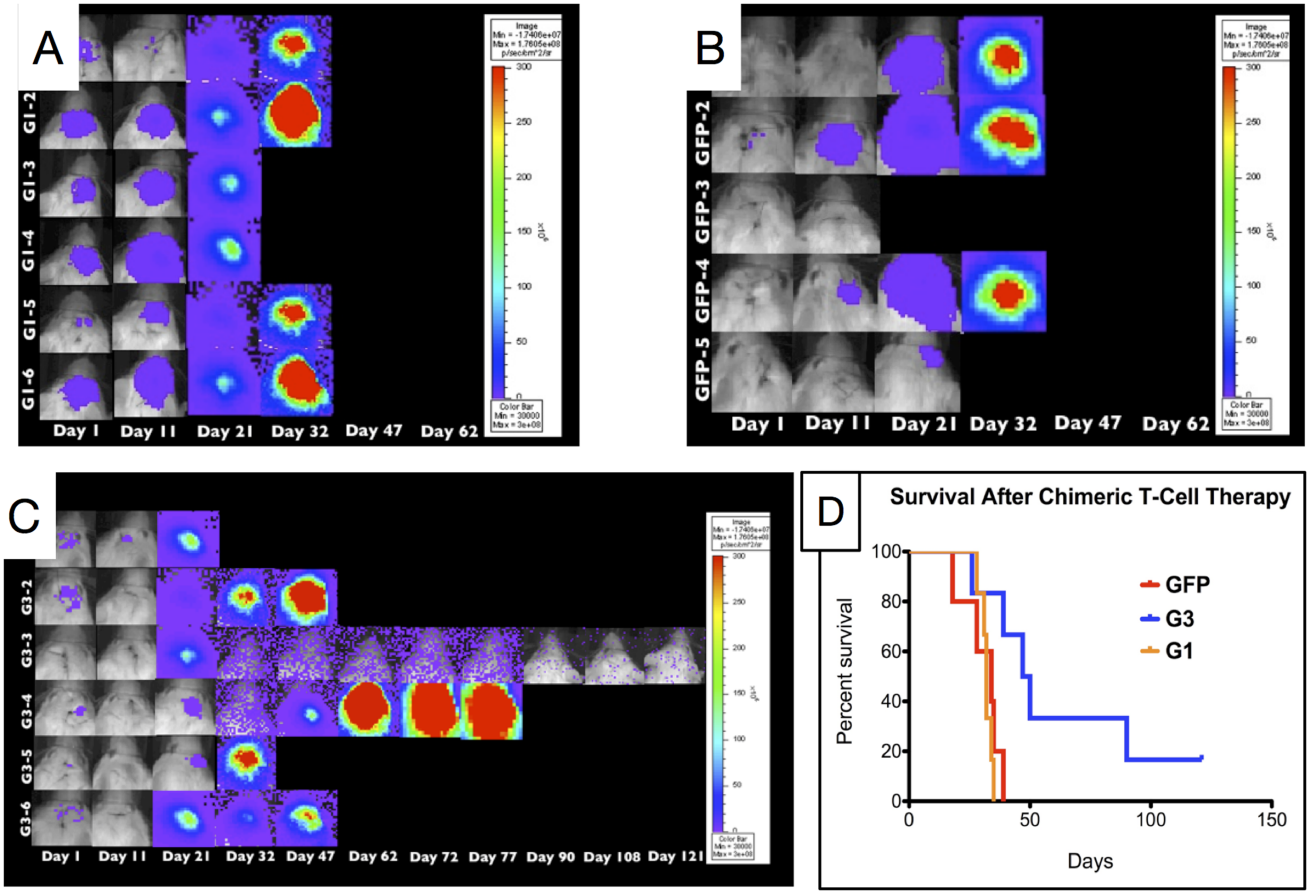
*In vivo* activity of G3-T-cells in an intracranial glioblastoma model. (A-C)25,000 firefly luciferase expressing U87vIIIffluc human glioblastoma cells were mixed with 25,000 G1, G3, or GFP-T-cells (1:1) immediately before intracranial implantation into the forebrain of NOD-scid mice. Starting form day 1, bioluminescence was measured every 3-5 days for 121 days using Xenogen living image system (IVIS^®^, Xenogen). Prior to imaging, animals were anesthetized and luciferin substrate was administered. Living image^®^software (Xenogen) was used for imagind and signal quantification. (D)Kaplan-Meier survival curve for single, low-dose treatment cohort.

The cell proliferation and IFN-*γ* expression was highest in G3-EGFRvIII CAR T-cells cocultured with CD32-80-137L-EGFRVIIIΔ654 aAPC cells. We therefore nucleofected freshly harvested PBMCs with G3-EGFRvIII CAR, G1-EGFRvIII CAR, or PMG-GFP plasmids and co-cultured with CD32-80-137L-EGFRVIIIΔ654 aAPC feeder cell line until fourth restimulation cycle to assess their activity *in vivo*. Prior to the stereotactic injection of T-cells, we mixed each T-cell groups with the U87vIIIffluc target cell line. *In vivo* bioluminescence images (BLI) revealed a prolonged survival in G3 mice compared to G1 and GFP groups (Fig 6). The maximum BLI value of G1 group was observed as 87,781x10^6^ photons per second (photons/sec) at day 32, 11,432x10^6^ photons/sec at day 35 for GFP, and 16,068x10^6^ photons/sec at day 47 for G3 groups. Two mice of the G3-EGFRvIII CAR group survived as long as 90 and 121 days (Fig 6C), and the mean survival of G3-EGFRvIII CAR, G1-EGFRvIII CAR, GFP, and groups was 48.5, 32, and 34 days respectively (Fig 6D).

**Fig 7:**
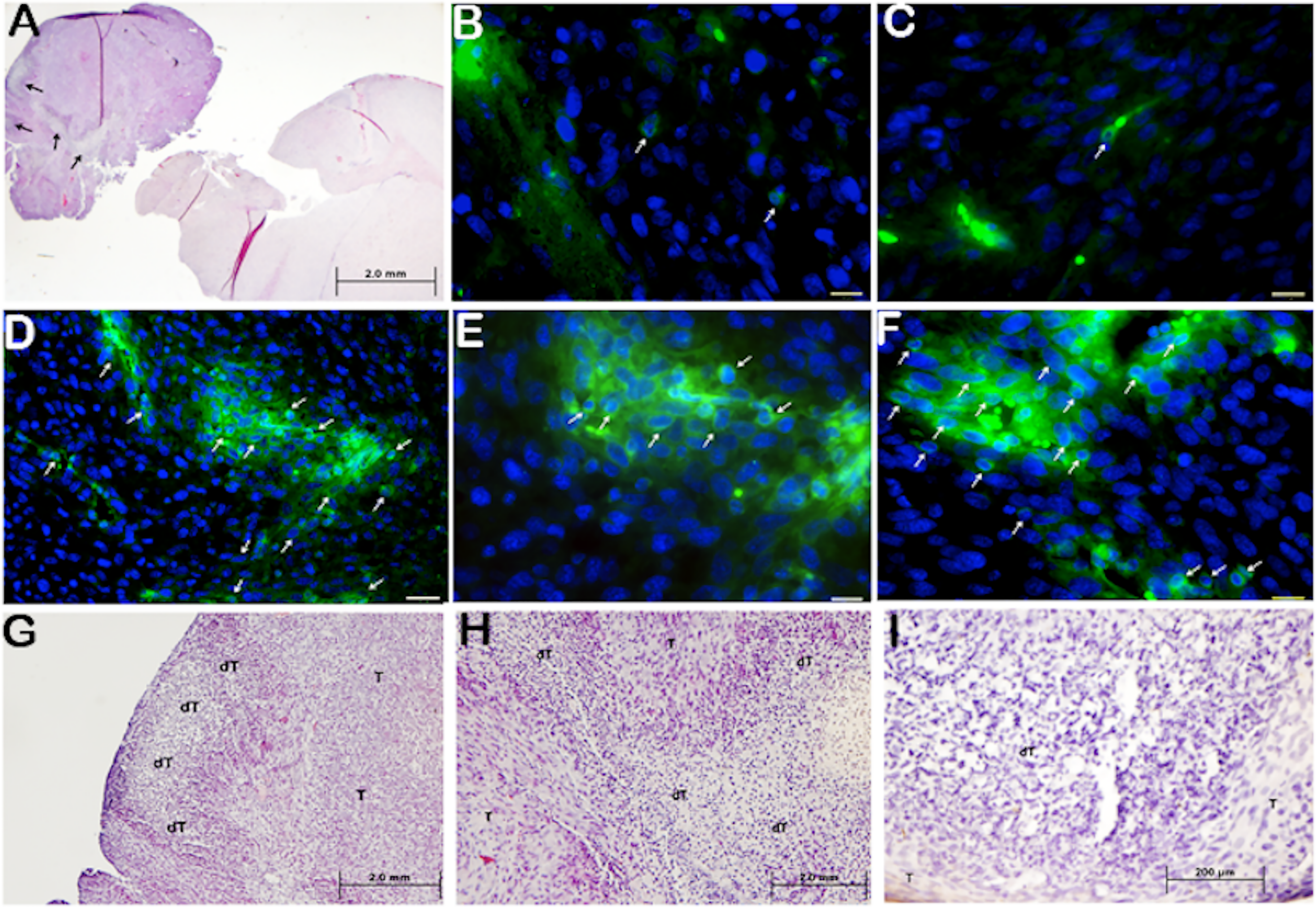
Histological analysis of T-cell persistence. (A)Representative hematoxylin and eosin sections are presented for G3 mouse prior to natural death at day 90 (A, G-I). (B-F)Representative immunofluorescence anti-human CD3 brain histology images of tumor bed of G3 (D-F), G1 (B), or GFP-T (C) cell treated mouse groups prior to natural death at day 90 (G3), or 35 days (G1 and GFP) after initial tumor and T-cell grafting. G3 tumor tissue was pictured at 200x (D) and 400x of the same area (E), and at 400x of a different tumor area of the same tissue (F). Pictures are shown the demolished tumor tissue disconnected to tumor bed at 20x magnification (D). Same tumor tissue was pictured at 100x (G), 200x (H) and 400x (I) magnification, demonstrating demolished tumor tissue (dT) within the tumor bed (T).

To further elucidate hypothesized T-cell survival *in vivo*, we performed CD3 staining on mouse brain sections as well as generated H&E reference sections. Results indicated that in all mouse brain sections of G1-EGFRvIII CAR or GFP groups that survived until maximum of day 35, only rare T-cells were found to be persistent in the tumor area. However, in all of the 4 mice brain sections of G3-EGFRvIII CAR group, one of which survived the longest term until day 90 (Fig 7A and 7D–7I), we identified multiple T-cell clusters at several tumor areas.

Under microscopic evaluation of H&E sections, we also identified focal destruction of regions of connective tissue within tumor stroma (Fig 7D–7F). The coupling of findings of persistent clusters of T-cells at day 90 and tumor associated tissue destruction, suggested that G3-EGFRvIII CAR T-cells can not only survive in extended periods compared to G1-EGFRvIII CAR and GFP-T-cells, but also actively destroy tumor cells in a single treatment. In none of the G1-EGFRvIII CAR of GFP groups was such tissue destruction observed.

## Discussion

In this study, we present a ‘third generation’ anti-EGFRvIII CAR, and EGFRvIII specific artificial antigen presenting cells (aAPCs) for ex-vivo target-specific activation and T-cell persistence *in vivo*. We assessed the survival and efficiency of G3-EGFRvIII-CAR-T cells in long-term cultures and *in vivo* when cocultured with CD32-80-137L-EGFRVIIIΔ654 aAPCs. Our results show the survival of G3-EGFRvIII CAR T-cells within the tumor as long as 90 days after a low-dose treatment and single administration without any need for ongoing IL-2 supplementation, accompanied by a marked tumor stroma demolition. CAR T-cell therapies have demonstrated significant clinical success in the treatment of CD19+ hematological malignancies, with complete response rates reported for the majority of patients with acute lymphoblastic leukemias and follicular lymphoma [7, 26] [and American Society of Clinical Oncology, ASCO 2015]. A major goal for the field of CAR T-cell therapies is its successful application to solid tumors. There are several barriers to overcome to achieve this goal, with CAR design and T-cell manufacturing being critical components for maximal therapeutic success [17]. Further important criteria include the tumor antigen selection, overcoming the immunosuppressive tumor microenvironment and T-cell persistence, and safety considerations [6, 17]. Here, we present the development of reagents for the production of a ‘third generation’ of CAR that is EGFRvIII-specific, expressing CD28 and OX40 intracellular costimulatory domains for T-cell persistence, and new antigen-specific artificial antigen presenting cells (aAPCs) for target-specific ex-vivo activation and culture improvement.

Most antigens targeted by CARs are not tumor “specific”, but tumor “associated”, meaning that although tumors may express higher masses of these antigens, they are also expressed in normal tissues in the body. Targeting such antigens may cause significant off-target toxicities in patients treated with CARs that are not truly tumor specific [8, 27]. Up to 30% of GBM specimens express the EGFRvIII mutant protein, which is targeted by G3-EGFRvIII CAR cells in this study. Because this mutant protein is not expressed in normal brain, it presents itself as an attractive target for GBM therapies including adoptive CAR T-cell therapy as described here and in ours and other’s previous studies [2, 4, 13–16].

We have generated a ‘third generation’ EGFRvIII-specific chimeric immune receptor that is engineered to signal through endodomains composed of CD28-OX40-CD3*ζ* (G3-EGFRvIII CAR). We compared this construct with our original, ‘first generation’ EGFRvIII-specific CAR, which signals through the intercellular domain of CD3*ζ* (G1-EGFRvIII CAR) [2]. CAR constructs were stably expressed by primary T-cells using plasmid nucleofection method and by hygromycin drug selection. As expected, we have observed similar cytolytic potential of both G1 and G3-EGFRvIII CARs upon co-incubation with target cells *in vitro*. This is consistent with the results of other studies that have shown no difference in tumor-specific *in vitro* cytolysis in engineered CARs that either include or lack costimulatory signals [28–30].

Rapid CAR T-cell expansion is an important parameter for adoptive immunotherapy in clinical settings. This provides a challenge particularly in non-viral-based CAR systems, where long culture periods (up to three months) are required to obtain the necessary yield of CAR T-cell products. Therefore, the development of culture methods that provide long-term expansion conditions, which maintain a ‘younger’ T-cell phenotype for active killing properties becomes critical [17].

Here, we have shown that in both short-term and long-term (6th restimulation cycle for 42 days) culture periods, G3-EGFRvIII CAR T-cells maintain their active killing property when cocultured with CD32-80-137L-EGFRVIIIΔ654 aAPCs. Using the K562 cell line as artificial antigen presenting cells has some benefits: 1) these cells do not express major histocompatibility complex molecules, thus preventing allogenic reponses, and 2) they also express adhesion molecules that enhance interactions between T-cell and aAPC [23]. It was previously shown that K562 expressing ligands for both CD28 and CD137 (4-1BB) and the additional inclusion of CD80 are superior for long-term propagation of CD8 T-cells, and provided a superior expansion thereof [23]. Indeed, our results show that our G3-EGFRvIII CARs demonstrated substantial expansion in short-term culture when exposed to aAPCs or EGFRvIII antigen, however, for longer term culture under selection, the CD32-80-137L-EGFRvIII Δ654 aAPC cells (expressing EGFRvIII, CD32, 80, and 41BBL) provided a superior cell expansion of G3-EGFRvIII CAR T-cells compared to other aAPCs. During long-term expansion under selection, there is significant negative growth pressure on the population of transduced T-cells. This results in a growth delay due to hygromycin selection, which was previously reported [31]. However, hygromycin resistant CAR T-cells have been used in clinical trials with no accompanied toxicity to patients [31, 32]. Importantly, G3-EGFRvIII CAR T-cells maintained their proliferative capacity and cytolytic function after long-term culture conditions or multiple restimulation cycles. This observation fits well with Pule and colleagues study, in which retrovirally transduced T-cells expressing CD28-OX40-CD3*ζ* provided a prolonged survival and augmented cytokine release [21].

Research has shown that even in the absence of added IL-2, CD28 and OX40 an enhanced specific cytolysis [33]. Comparable to these results, we found that without addition of IL-2, IFN-*γ* release was increased in G3-EGFRvIII CAR T-cells, cocultured with EGFRVIIIΔ654 aAPCs, CD32-80-137L-EGFRVIIIΔ654 aAPCs, or CD32-80-137L aAPCs after specific cytolysis. Interestingly, we observed increased IFN-*γ* release in ‘first generation’ EGFRvIII-specfic T-cells consisting of CD3*ζ* signaling (G1-EGFRvIII CAR) when cocultured with CD32-80-137L aAPCs. CD32-80-137L aAPCs express ligands for T-cell receptor (TCR) and the CD28 and 4-1BB costimulatory surface molecules. It is reported that these aAPCs activate and rapidly expand polyclonal or antigen-specific CD8 (+) T-cells [34]. Involvement of such ligands and costimulatory molecules may be involved in enhanced antigen-specific lysis by G1-EGFRvIII CAR T-cells when cocultured with CD32-80-137L aAPCs. However, antigen-specific aAPCs such as CD32-80-137L-EGFRVIIIΔ654 or EGFRVIIIΔ654 aAPCs increased antigen-specific lysis only in G3-EGFRvIII CAR T-cells. Elevated levels of antigen-specific cytokine release in G3-EGFRvIII CAR T-cells when cocultured with EGFRVIIIΔ654 aAPCs may suggest that G3-EGFRvIII CAR T-cells indeed overcome antigen-induced cell death (AICD) due to its integrated co-stimulatory molecules. Hombach and Abken have reported that OX40 signaling was most efficient in preventing activation induced cell death of effector memory T-cells over CD28 signaling [33]. Furthermore, CD28 was superior to initiate the T-cell response, whereas OX40 was most efficient in sustaining long-term response. It is not clear however, whether TCR ligands and costimulatory surface molecules on CD32-80-137L-EGFRVIIIΔ654 aAPCs are in competition with the antigen molecule on the aAPC surface.

Our results show lower levels of antigen-specific IL-2 production. Wilkie and colleagues have reported low IL-2 production by dual CAR T-cells, while reason remains unknown [35]. It has been shown that administration of IL-2 at low dose prolonged the *in vivo* persistence of CAR-targeted T-cells [36]. However, IL-2 is toxic when administered in high doses, mediated in part through the induction of systemic autophagy [37]. Furthermore, it is known that IL-2 has *in vitro* and *in vivo* immunoregulatory functions and can exert its immunosuppressive functions by stimulating the generation and homeostatis of regulatory T-cells (Treg), and can induce activation-induced cell death (AICD) [38]. Therefore, lower IL-2 expressing CARs may represent ideal tools for clinical use.

Previous research has shown that addition of OX40 did not increase cytolytic activity [33]. In accordance of this finding, we have found no increase in *in vitro* cytolytic activity by addition of CD28 and OX40 costimulatory molecules in MR1 CARs. However, we demonstrated that G3 CAR T-cells (CD28-OX40-CD3*ζ*) produced long-lasting anti-tumor activity in an orthotopic xenograft mouse model. Yvon et al have shown that a CD28-OX40 CAR provided lasting anti-tumor activity in a xenograft metastatic melanoma model. Though disease was not completely eradicated, mice were alive at day 100 [39]. In this model, 1x10^7^ intravenous CAR T-cells were injected twice over the period of treatment. Here, we have shown that G3 CAR T-cells, cocultured with CD32-80-137L-EGFRVIIIΔ654 aAPC cells (CD32, CD80, 41BBL, and EGFRvIII) can survive at day 90, provide enhanced anti-tumor activity, and able to destroy tumor stroma with single treatment of 25,000 CAR T-cells. Coculturing with CD32-80-137L-EGFRVIIIΔ654 aAPC cells may bring additional advantage for enhanced anti-tumor activity due to additionally providing antigen-specific stimulation. Combining two strategies of using EGFRvIII-specific G3 CAR T-cells cocultured with antigen specific and costimulatory molecule producing aAPCs might therefore represent a potent therapeutic approach for GBM.

There is ongoing concern about using viral vectors for CAR transfectants for therapeutic purposes [20], hence, technically, those vectors provide short-term transfectants, thus requiring multiple administrations of the CAR T-cells as therapeutic agent. However, the naked DNA method of generating CAR T-cells has several benefits over a viral system as well as provides several clinical benefits, such as 1) safety for therapeutic uses, 2) it generates long-term stable transfectants, 3) the production is cheaper. There are concerns on the possible terminal differentiation or senescence of long-term cultures of CAR T-cells [40, 41]. In the described system here, we have shown here that using antigen specific designer aAPCs can both shorten the length of culturing period, and retain effective target cell lysis after multiple (up to 6) restimulation cycles. While this approach utilized hygromycin selection for longer term cultures as previously described in Phase 1 studies, less toxic selection drugs such as Geneticin may be suitable as such systems have been used to select and expand retrovirally transduced T-cells for ovarian cancer in a Phase I study [42].

Of note, this CAR system consists of an integrated HSV-TK suicide gene system for potential clinical studies in human. Although other suicide gene systems such as iCasp9 [43], and CD20 [44] have been found to be effective in destruction of transduced T-cells [45], a “one size fits all” suicide gene is yet to be identified.

Little is known about CAR T-cell activation and its behavior in the tumor microenvironment. The common assumption of CAR T-cell activation mechanism is that after activation of CAR T-cells, tumor cells can be eliminated directly by the CAR T-cells or other immune cells be recruited to the tumor microenvironment [46]. In the present orthotopic xenogenic human glioblastoma model in NODScid mice tumor stromal degradation by G3 CAR T-cells at day 90 after initial implant may occurred directly by G3-T-cells. Since incorporation of additional signaling domains may be able to bypass some of the restrictions of immunosuppressive cells, such as Tregs, or immunosuppressive cytokines secreted by tumor cells, or amplify effects of recruitment of other immune cells to the tumor microenvironment [46], in depth analysis of the effect of G3 CAR T-cells in clinical studies would provide valuable insights on its mechanism of activation in gliomas.

Another important factor to consider for effective T-cell persistence and increased anti-tumor responses is the phenotype of T-cells. Both CD8+ and CD4+ T-cell subsets are required for potent and sustained anti-tumor response [47]. Our methodology of using a bulk population of PBMCs might be therefore beneficial in sustaining both CD8+ and CD4+ populations compared to CD8+ T-cell isolation prior to the CAR transfer. We have observed an increased CD4+ population in G3 CAR T-cells after 6th restimulation cycle for 42 days, co-cultured with CD32-80-137L-EGFRVIIIΔ654 aAPC cells (data not shown). Whether or not the integration of the OX40 costimulatory domain has an effect in the increase of CD4+ population, or whether the *in vivo* anti-tumor response is CD4+ dependent remain to be elucidated.

Maher suggests administration of smaller CAR T-cell doses for of safety and practicality, development of T-cells to expand *in vivo* in a controlled manner, which persist *in vivo* for longer [48]. With this study, we believe to have accomplished these important criteria in adoptive immunotherapy by showing EGFRvIII-specific ‘third generation’ EGFRvIII CAR T-cell potency and long-term anti-tumor effect in a xenogenic human glioblastoma model. Due to high expression of EGFRvIII in GBM tumors, we believe that this therapy strategy may complement standard treatment and care, such as radio and chemotherapy for patients suffering from this disease.

## Conclusion

In summary, we have developed an EGFRvIII-specific third generation (G3-EGFRvIII) chimeric antigen receptor (CAR) that expresses both co-stimulatory factors CD28 and OX40 (MR1-CD8TM-CD28-OX40-CD3*ζ*). We further generated artificial antigen presenting cell lines (aAPC) expressing the extracellular and transmembrane domain of EGFRvIII (EGFRVIIIΔ654) with costimulatory molecules including CD32, CD80 and 4-1BBL (EGFRVIIIΔ654 aAPC and CD32-80-137L-EGFRVIIIΔ654 aAPC). We show the persistence of G3-EGFRvIII-CAR-T cells in long-term cultures and *in vivo* when cocultured with CD32-80-137L-EGFRVIIIΔ654 aAPCs. In addition, and importantly, we observed survival of G3-EGFRvIII CAR T-cells within the tumor as long as 90 days after implantation after a low-dose treatment and single administration without any need for ongoing IL-2 supplementation, accompanied by a marked tumor stroma demolition. Our findings suggest that G3-EGFRvIII CAR in combination with CD32-80-137L-EGFRVIIIΔ654 aAPCs warrants itself as a potential anti-tumor therapy strategy for glioblastoma.
